# Deep learning-based automatic field of view planning for prostate MRI in oblique coronal and oblique axial planes

**DOI:** 10.1038/s41598-026-52248-6

**Published:** 2026-05-11

**Authors:** Anton Sheahan Quinsten, Axel Wetter, Maciej Raczkowski, Łukasz Trembecki, Tomasz Guz, Samuel Oliveira, Rüdiger Buchkremer, David Matusiewicz, Kai Nassenstein, Michael Forsting, Aydin Demircioğlu

**Affiliations:** 1 Institute of Diagnostic and Interventional Radiology and Neuroradiology, University Hospital Essen, Hufelandstraße 55, 45147 Essen, Germany; 2 Asklepios Clinic Hamburg, Hamburg, Germany; 3 Department of Oncology, Wroclaw Medical University, pl. L. Hirszfelda 12, Wroclaw, 53-413 Poland; 4 Micro Solutions, Sp. z o.o, ul. Legnicka 55f, Wrocław, 54-203 Poland; 5 Everything MRI, London, UK; 6 Institute of IT Management and Digitization Research (ifid), FOM University, Düsseldorf, Germany; 7 Institute for Health & Social Science (ifgs), FOM University, Essen, Germany

**Keywords:** Prostate cancer, Prostate MRI, Field of view, Deep Learning, automation, Health care, Medical research

## Abstract

Accurate field-of-view (FoV) prescription in oblique coronal and axial planes is essential for high-quality prostate MRI but remains operator-dependent and variable. We developed and evaluated a ResNet-based deep learning framework for automated FoV planning. In this retrospective multicenter study, FoV prescriptions were annotated on PI-CAI dataset. Three readers assessed intra- and inter-rater variability to establish reference consistency. Three neural network variants were trained on 1,474 examinations from PI-CAI dataset (2012–2021), and the optimal model was selected by internal validation. Generalizability and clinical utility were tested on three external cohorts totaling 530 examinations (2021–2024) using a non-inferiority design. The selected model achieved non-inferior performance for slice positioning, with differences ranging from 0.16 ± 0.99 to 0.37 ± 0.48. Across sites, FoV overlaps ranged from 82.4 ± 4.1% to 88.7 ± 6.0%, and the angle differences between predicted and reference planes were 4.66 ± 4.89° (Site I), 3.46 ± 2.80° (Site II), and 2.99 ± 2.90° (Site III). Clinical utility was high at all sites, with acceptability rates of 97.9%, 97.7%,98.8%, 98.1% and 98.1% for Site I (Raters 1–5), 95.7%, 97.8%, 100%, 95.7% and 97.8% for Site II (Raters 1–5), and 100% for all raters at Site III. These findings demonstrate the feasibility of automated FoV positioning for prostate MRI and indicate excellent clinical utility.

## Introduction

Prostate cancer (PCa) is the second most common cancer diagnosis and the fifth leading cause of mortality in men worldwide^1^; furthermore, it is expected to increase by 85% by 2040.^2^.

**Fig. 1:**
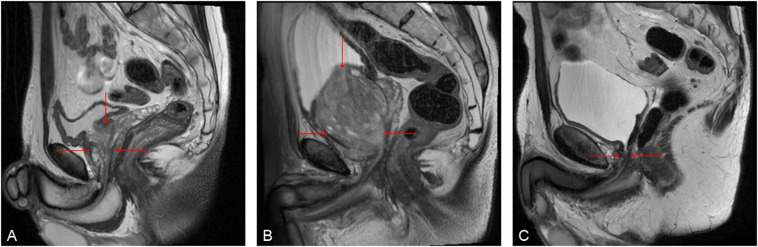
Representative T2-weighted sagittal prostate MR images illustrating variable complexity across different anatomical and postoperative conditions. (**A**) Normal prostate with regular gland size and preserved zonal anatomy, representing a standard scenario. (**B**) Pathologically enlarged prostate with increased gland volume and altered anatomical extent. (**C**) Post-enucleation appearance with marked postoperative alteration of the prostate bed, representing a particularly challenging scenario due to missing anatomy.

Prostate MRI has emerged as the foremost non-invasive imaging technique for diagnosing and managing clinically significant prostate cancer (csPCa) (Fig. 1), and offers high sensitivity, as demonstrated by evidence-based studies such as PROMIS^3^ and PRECISION^4^. It also provides valuable insights into local tumor staging^5^, facilitates active surveillance strategies^6^, and serves as a viable screening examination^7^. However, the efficacy of prostate MRI in detecting PCa depends on the quality of the acquired images^7,8^. Inadequate image acquisition has been associated with diminished accuracy, heightened uncertainty, and increased biopsy procedures with lower cancer yield^9^.

**Fig. 2:**
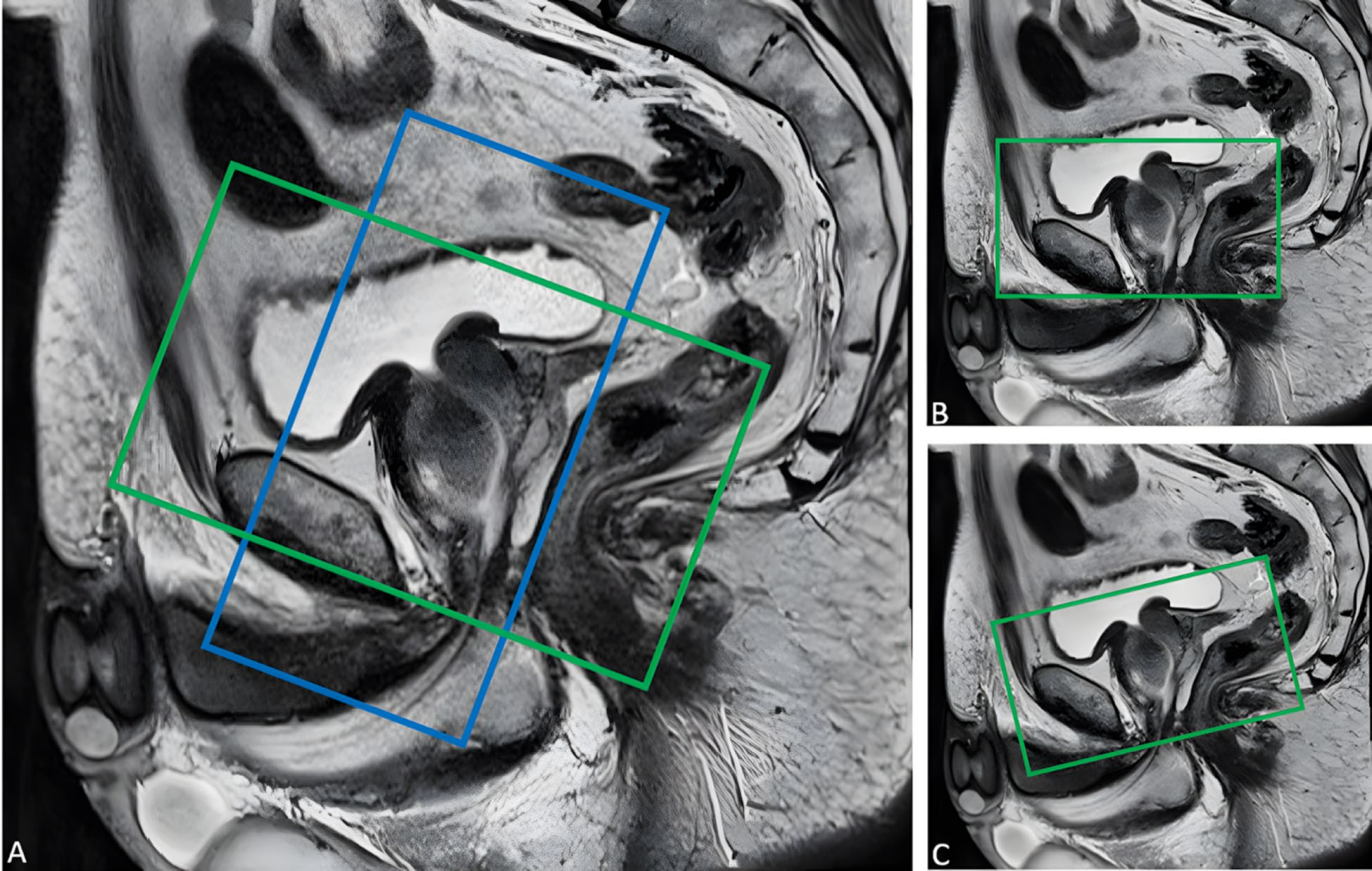
Three variants of the axial field of view (FoV) according to PI-RADS: first variant (green box, A), oblique axial plane orthogonal to the prostate-rectal interface; second variant (green box, B), straight axial to the patient; and third variant (green box, C), oblique axial plane orthogonal to the long axis of the prostate. The oblique coronal plane parallel to the prostate-rectal interface is also displayed (blue box, A).

To standardize image acquisition and data interpretation, the European Society of Urogenital Radiology (ESUR) established the Prostate Imaging Reporting and Data System (PI-RADS)^10^. Here the acquisition of high-resolution T2-weighted imaging (T2WI) scans in the axial plane, either directly axial to the patient or in an oblique plane aligned with the long axis of the prostate or orthogonal to the rectum, is recommended (Fig. 2)^11^. However, given that exact positioning and correct angulation are crucial for an accurate diagnosis, further studies have been conducted on how the field of view (FoV) should be positioned and which pitfalls to consider^12,13^. Engels et al. proposed that the oblique coronal planning should be aligned parallel to the prostate-rectal interface, presenting the prostate in a “heart shape,” and that the axial planning should be perpendicular and orthogonal to the rectum. This alignment optimizes visualization of tumor extension to the seminal vesicles and facilitates the comparison between whole-mount radical prostatectomy slices and axial T2WI scans^13^.

Prostate MRI is generally performed according to manual prescriptions by specially trained radiographers. Manual FoV prescription in MRI is time-consuming^14,15^, susceptible to error and inconsistency^16^ and characterized by limited reproducibility due to inter-rater variability^17^. This can lead to poor overall image quality, and the resulting scans can exhibit artifacts, noise, and a low signal-to-noise ratio (SNR), potentially leading to repeat examinations. The image quality is contingent upon patient related factors, scan acquisition factors and the knowledge and experience of the examining radiographer^18^.

Automatic FoV planning could ensure reproducibility in the diagnosis, affording fast and accurate positioning with less variance across patients or within longitudinal scans of the same patient^19^. Recent years have seen notable advancements in deep learning (DL) for prostate MRI, which has been reported to automate tasks such as assessing the scan quality^20^, segmentation^21^ and lesion detection^22^. DL could, therefore, present a solution for the automatic planning of the FoV for prostate MRI.

To address this gap, we developed a ResNet-based framework that predicts patient-specific FoV position, extent, and orientation in the oblique coronal and axial planes. We benchmarked network variants and conducted internal validation and external multicenter testing, evaluating performance using slice-position error, intersection over union (IoU), mean absolute angle difference (MAAD), center offset, and Hausdorff distance (HD), alongside blinded reader assessments of clinical utility and non-inferiority analyses versus expert prescriptions. This study establishes a DL-based solution for fast, accurate, and reproducible FoV planning in prostate MRI.

## Results

### Patient cohorts

**Fig. 3:**
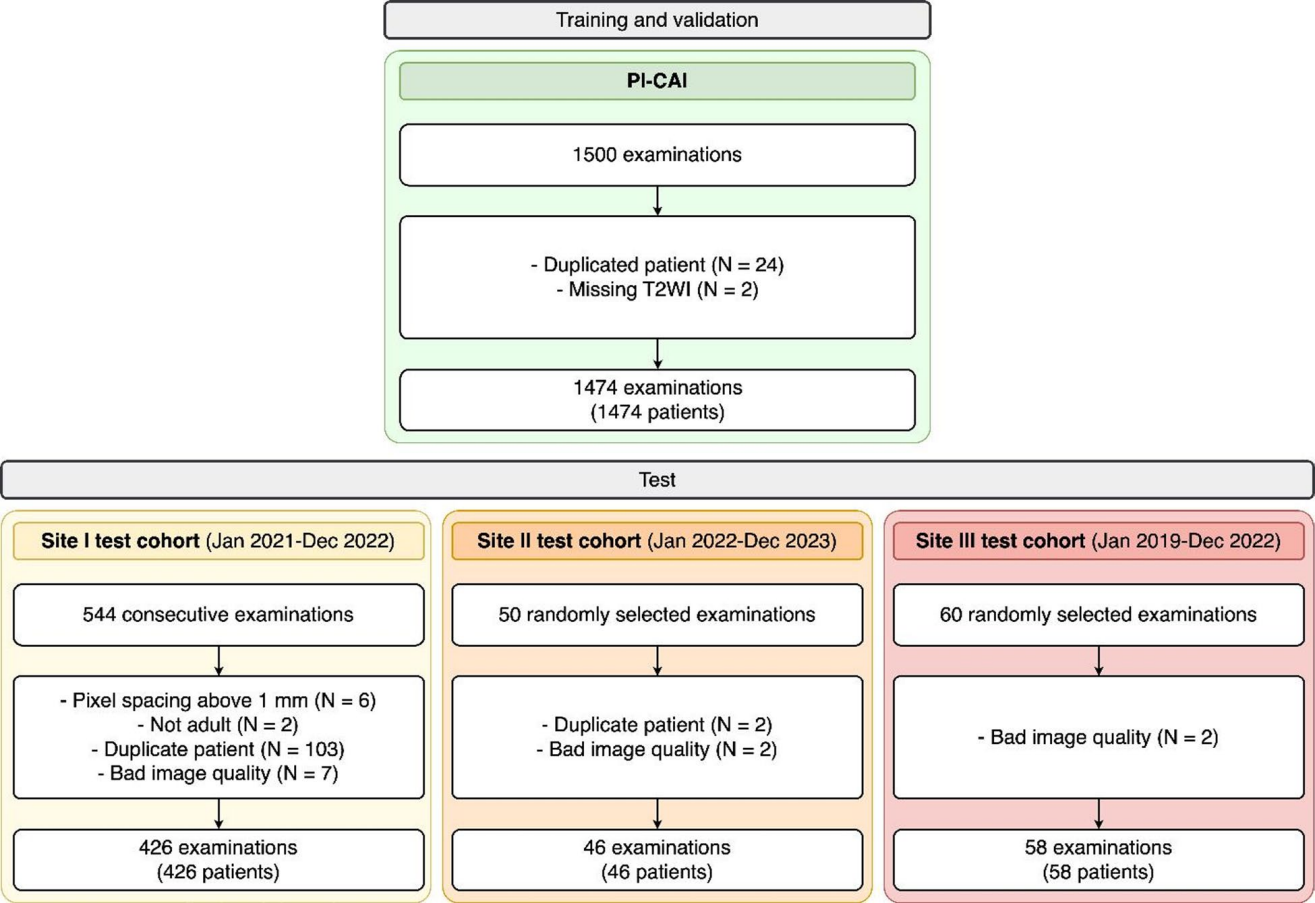
Patient flowchart.

A total of 1474 prostate MRI scans were included for training and 530 for external testing (Fig. 3; Table 1). The mean age was 65.6 ± 7.2 years for the training patients, 66.0 ± 8.6 years for the Site I test cohort (*P* = 0.34), 69.4 ± 8.4 years for the Site II cohort (*P* < 0.001), and 62.1 ± 10.0 years for the Site III cohort (*P* = 0.001).

**Table 1:** Scanners used in the study.

Scanner	All (*N* = 2004)	Train (PI-CAI) (*N* = 1474)	External Test (Site I) (*N* = 426)	External Test (Site II) (*N* = 46)	External Test (Site III) (*N* = 58)
Siemens MAGNETOM Skyra (3T)	1371	1020	351	0	0
Philips Ingenia (1.5T)	225	217	0	8	0
Siemens Avanto (1.5T)	26	13	13	0	0
Philips Achieva (1.5T/3T)	90	52	0	38	0
Siemens MAGNETOM Prisma (3T)	88	88	0	0	0
Siemens MAGNETOM Trio (3T)	67	67	0	0	0
Siemens Biograph (3T)	32	0	32	0	0
Siemens Aera (1.5T)	26	17	9	0	0
Siemens MAGNETOM Vida (3T)	21	0	21	0	0
GE Insignia (3T)	58	0	0	0	58

### Imaging data

The T2WI sagittal plane used in the training set had a voxel size of 0.5 × 0.5 × 3.0mm^3^ in most cases; for the test cohorts the voxel size was between 0.3 × 0.3 × 3mm^3^ and 0.7 × 0.7 × 4mm^3^ for Site I, between 0.5 × 0.5 × 3mm^3^ 0.5 × 0.5 × 4.0mm^3^ for Site II, and 0.5 × 0.5 × 3.5mm^3^ for Site III. The variance in repetition time across the three test cohorts ranged from 2940ms to 8060ms, while the variance in echo time was from 78ms to 169ms (Table 2). The number of slices varied between 15 and 31 in the training dataset, and 19 and 35 (Site I), and 12 and 26 (Site II), and 23 and 25 (Site III) in the test cohorts with different gaps.

**Table 2:** Protocol parameters.

	TR (ms)	TE (ms)	Slice thickness (mm)	Matrix size	Inplane resolution (mm^2^)
Site I					
1.5T	3020–6630	83–112	3–4	270–512	0.3 × 0.3–0.7 × 0.7
3T	2940–8060	78–126	3–4	320–640	0.3 × 0.3–0.6 × 0.6
Site II					
1.5T	3480–4254	120	4	432	0.5 × 0.5
3T	3680–4907	96	3	336	0.5 × 0.5
Site III					
3T	4996–6698	156–169	3.5	384	0.5 × 0.5

### Intra- and inter-rater variability

Overall, the intra-rater variability was lower than the inter-rater variability (Table 3). The annotations of the two raters involved in the ground truth annotation were closer to each other than to the annotation of the third rater, who was independent. This effect was seen across all measurements.

**Table 3:** Results of the intra- and inter-rater variability assessments. To account for pathological variations in prostate size, center offset and HD95 are reported as percentages relative to the longer side of the ground-truth FOV dimensions. HD95 = 95th percentile Hausdorff Distance.

Slice difference				
	Rater 1		Rater 2	Rater 3
Rater 1	0.11 ± 0.31		-	-
Rater 2	0.15 ± 0.36		-	-
Rater 3	0.6 ± 0.77		0.61 ± 0.79	-
**IoU (%)** (oblique coronal FoV=lower triangle; oblique transversal FoV=upper triangle)				
Rater 1	88.97 ± 7.93%	86.65 ± 7.95%	86.09 ± 8.57%	71.37 ± 8.56%
Rater 2	87.4 ± 8.72%		-	69.76 ± 6.96%
Rater 3	71.96 ± 7.18%		73.66 ± 7.99%	-
**Angle difference (**°) (oblique coronal FoV=lower triangle; oblique transversal FoV=upper triangle)				
Rater 1	1.94 ± 3.31°	1.96 ± 3.3°	2.57 ± 4.08°	5.01 ± 4.42°
Rater 2	2.54 ± 4.09°		-	5.12 ± 4.8°
Rater 3	5.03 ± 4.42°		5.09 ± 4.81°	-
**Center offset (%)** (oblique coronal FoV=lower triangle; oblique transversal FoV=upper triangle)				
Rater 1	2.85 ± 2.22%	3.15 ± 2.6%	3.15 ± 2.43%	10.45 ± 4.32%
Rater 2	2.73 ± 1.93%		-	11.2 ± 3.69%
Rater 3	6.42 ± 2.07%		5.97 ± 2.36%	-
**HD95 (%)** (oblique coronal FoV=lower triangle; oblique transversal FoV=upper triangle)				
Rater 1	4.63 ± 3.99%	5.99 ± 4.3%	6.29 ± 4.59%	14.45 ± 4.89%
Rater 2	5.25 ± 4.79%		-	15.27 ± 4.48%
Rater 3	11.52 ± 4.09%		11.2 ± 4.77%	-

### Neural network training

In the first stage, the three networks performed similarly during the CV, with only slight differences (Table 4). The optimal network was the Rotated RetinaNet model trained using a learning rate of 0.02, with an average slice difference of 0.35 ± 0.54, less than the inter-observer variability (0.6 ± 0.77). Therefore, Rotated RetinaNet was selected as the best-performing model. The ReDet model performed best during the second stage, with an IoU of 86.1 ± 0.06% and an MAAD of 5.75 ± 12.93**°** (Table 4).

**Table 4:** Results of the cross-validation of the first- and second-stage networks.

First stage network			
Model	Best learning rate	Mean slice difference ± SD	
Rotated RetinaNet	0.02	0.35 ± 0.54	
ROI transformer	0.08	0.36 ± 0.53	
ReDet	0.08	0.36 ± 0.53	

Second stage network			
Model	Best learning rate	IoU ± SD (%)	MAAD ± SD (°)
ReDet	0.08	86.1 ± 0.06	5.75 ± 12.93
ROI transformer	0.04	85.3 ± 0.07	7.42 ± 14.18
Rotated RetinaNet	0.008	81.9 ± 0.10	7.96 ± 14.59

### Testing

**Fig. 4:**
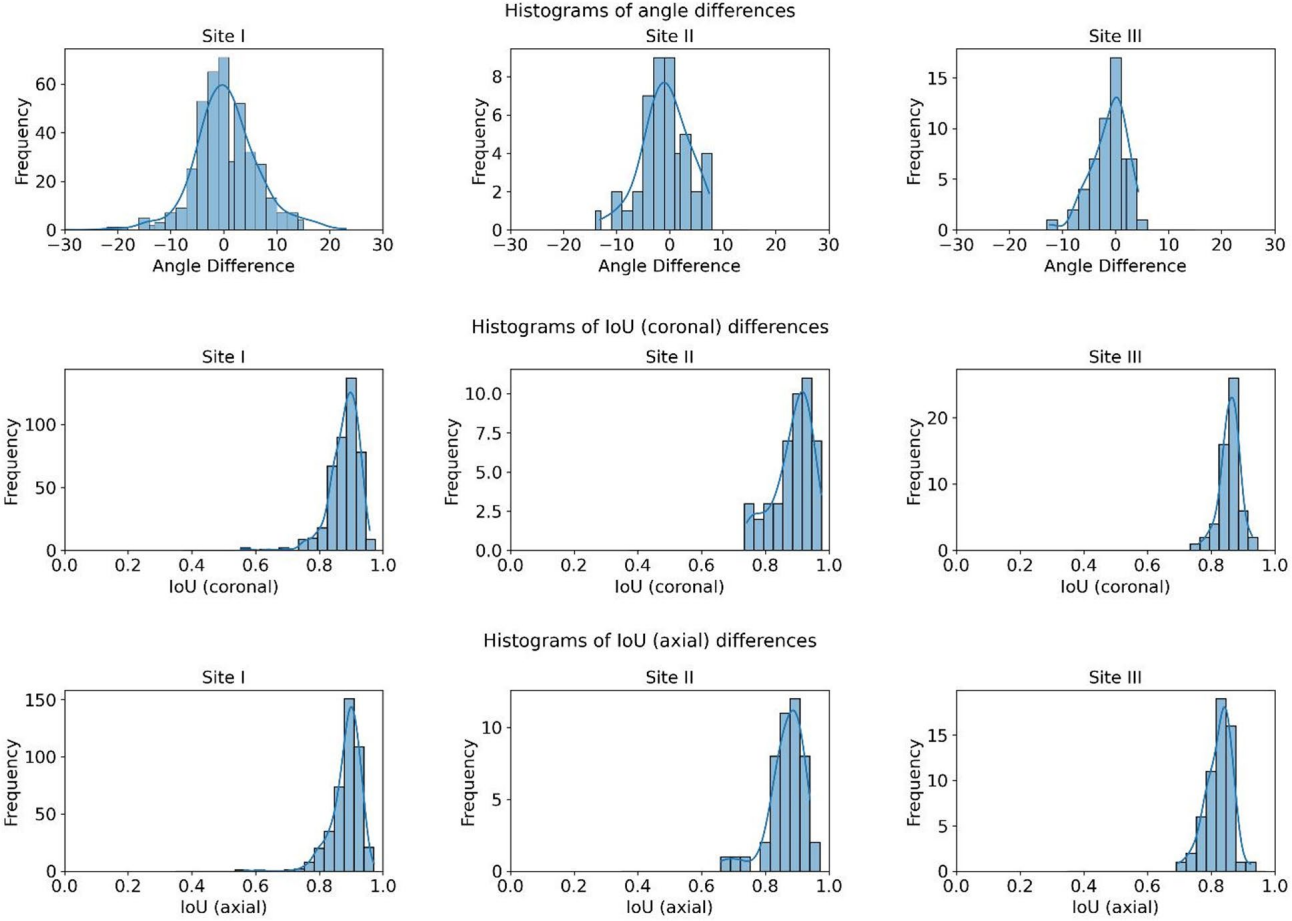
Histogram plots of the differences in the mean angle and Intersection-over-Union of the field-of-views between the ground-truth and network predictions for each site.

Overall, the performance on the test cohorts was close to that observed during the CV for Sites I, II, and III (Table 5; Fig. 4). Compared to the maximum inter-rater variability, the network predictions were almost all non-inferior across all sites, with the exception of MAAD at Site I and the 95th percentile Hausdorff distance at Site III (Table 5).

**Table 5:** Results from the independent test cohorts. The *P* values were obtained using a non-inferiority test against the maximum inter-rater variability. A significant *P* value means the corresponding annotations were not inferior to the maximum values observed in the inter-rater tests.

Test cohort	Mean slice difference ± SD	Oblique Coronal FoV IoU ± SD (%)	Oblique Axial FoV IoU ± SD (%)	MAAD ± SD (°)	Oblique Coronal FoV Center Offset (%)	Oblique Axial FoV Center Offset (%)	Oblique Coronal FoV HD95 (%)	Oblique Axial FoV HD95 (%)
Site I	0.36 ± 0.52 (*P* < 0.001)	78.7 ± 7.4 (*P* < 0.001)	79.6 ± 7.0 (*P* < 0.001)	4.66 ± 4.89 (*P* = 0.063)	3.78 ± 2.49 (*P* < 0.001)	3.31 ± 2.43 (*P* < 0.001)	11.09 ± 4.99 (*P* < 0.001)	11.04 ± 5.48 (*P* < 0.001)
Site II	0.37 ± 0.48 (*P* < 0.001)	80.2 ± 9.4 (*P* < 0.001)	76.7 ± 8.5 (*P* < 0.001)	3.46 ± 2.80 (*P* = 0.019)	4.10 ± 2.61 (*P* < 0.001)	3.56 ± 1.93 (*P* < 0.001)	9.88 ± 6.16 (*P* = 0.001)	11.12 ± 5.04 (*P* < 0.001)
Site III	0.16 ± 0.36 (*P* < 0.001)	75.4 ± 4.7 (*P* < 0.001)	70.3 ± 5.9 (*P* < 0.001)	2.60 ± 2.35 (*P* < 0.001)	3.28 ± 2.01 (*P* < 0.001)	5.25 ± 2.89 (*P* < 0.001)	13.60 ± 3.71 (*P* = 0.28)	14.95 ± 4.59 (*P* < 0.001)
Pooled	0.34 ± 0.51 (*P* < 0.001)	78.5 ± 7.4 (*P* < 0.001)	78.4 ± 7.6 (*P* < 0.001)	4.33 ± 4.59 (*P* = 0.003)	3.75 ± 2.45 (*P* < 0.001)	3.54 ± 2.51 (*P* < 0.001)	11.26 ± 5.06 (*P* = 0.006)	11.47 ± 5.48 (*P* < 0.001)

### Clinical utility

Overall, the average clinical utility of the predictions was outstanding, reaching 98.51%. (Site I: 98.12%, Site I: 97.40%, and Site III: 100%) (Table 6). In three cases, the network failed to predict a FoV, whereas in five cases, the predicted FoV was misplaced.

**Table 6:** Results from clinical utility assessment. Values in the clinical utility columns report the percentage of correct predictions, followed by error distribution in parentheses (n_total_; e_slice_, e_angular_, e_FoV_, e_miss_), The numbers in parentheses in the columns denote the total number of errors in the corresponding cohort.; The total error count n_total_ is decomposed into four categories: wrong slice selection (e_slice_), incorrect angulation (e_angular_), misplaced prediction (e_FoV_), missing predictions (e_miss_). For instance, Rater 1 judged nine total errors at Site I, comprising no case of wrong slice selection, one case of incorrect angulation, 5 cases of misplaced FoVs, and 3 cases of missing prediction.

Test cohort	Rater 1 Clinical utility (%) (number of errors)	Rater 2 Clinical utility (%) (number of errors)	Rater 3 Clinical utility (%) (number of errors)	Rater 4 Clinical utility (%) (number of errors	Rater 5 Clinical utility (%) (number of errors)
Site I	**97.9 (9; 0**,**1**,**5**,**3)**	**97.7 (10; 0**,**2**,**5**,**3)**	**98.8 (5; 0**,**0**,**2**,**3)**	**98.1(8; 0**,**0**,**5**,**3)**	**98.1 (8; 0**,**1**,**4**,**3)**
Site III	**95.7 (2; 1**,**0**,**1**,**0)**	**97.8 (1; 0**,**0**,**1**,**0)**	**100 (0)**	**95.7 (2; 0**,**1**,**1**,**0)**	**97.8 (1; 0**,**0**,**1**,**0)**
Site III	**100 (0)**	**100 (0)**	**100 (0)**	**100 (0)**	**100 (0)**

### Sensitivity analysis

**Fig. 5:**
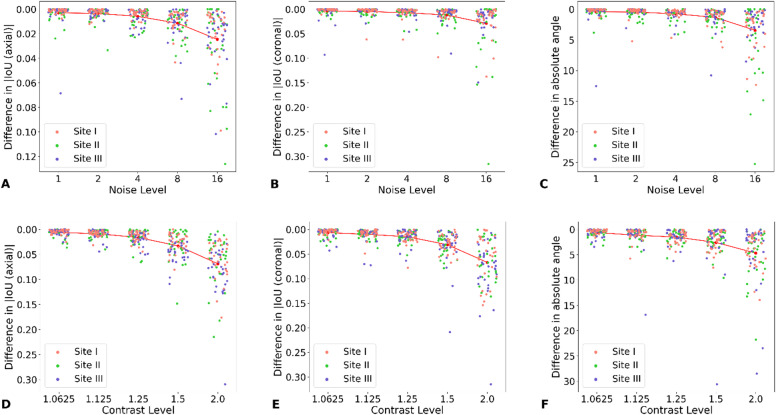
Robustness against increased noise and contrast. (A-C) Impact of changing the noise level of the selected slice on the Intersection-of-Union and angle of the FoVs. (D-F) Impact of changing the contrast level of the selected slice on the Intersection-of-Union and angle of the FoVs.

A visible dependence of prediction quality on both noise and contrast was observed (Fig. 5). Changes in contrast had a slightly smaller effect across the three test cohorts.

## Discussion

In this retrospective multicenter study, we demonstrated the feasibility of using DL to automatically position the FoV in the oblique coronal and axial planes for prostate MRI. Overall, the model-predicted and human-placed FoVs agreed. The clinical utility of the model-predicted FoV was confirmed by its high accuracy of 98.51% in three external cohorts.

DL-based automated FoV positioning offers significant clinical advantages including faster, accurate, and reproducible imaging, with reduced intra- and inter-rater variability compared to manual positioning^23–26^. The manual positioning of the FoV represents a challenge for radiographers unaccustomed to routine adherence to PI-RADS recommendations, leading to high variability in standardized FoV positioning and coverage^27,28^. Therefore automated and consistent FoV positioning for prostate MRI is crucial, focusing on its impact on reducing variability, enhancing reproducibility, and improving diagnostic accuracy, particularly in active surveillance and biopsy planning. Hence, we determined the ground truth with the aid of a radiologist and radiographer, both highly experienced in prostate imaging, through consensus. We employed three raters for intra- and inter-rater measurements to assess the variability. While the differences between the two raters involved in determining the ground truth were minor, the results of the third rater not involved in the ground truth determination exhibited larger differences. This discrepancy may be attributed to the lack of experience in prostate MRI of the third rater. Despite the low agreement, the slice difference was 0.6 ± 0.77, the IoU was 71.96 ± 7.18%, and the angle difference was 5.03 ± 4.42°, which does not affect the clinical utility.

The networks were trained in two stages: selecting the slice and annotating it. The training results of the three chosen network architectures in the first stage were excellent, exhibiting a mean slice difference of 0.36 ± 0.53, below the inter-rater variability of the third rater. This indicates that the network performs at a level comparable to that of a radiographer.

Subsequently, the most efficacious model was tested on three external cohorts. A high-performance level was achieved for the three centers with an overlap of 88% and an angle difference of 4.66 ± 4.89° (Site I), 3.46 ± 2.80° (Site II) and 2.6 ± 2.35° (Site III). These results are comparable to those achieved by the more inexperienced rater. Furthermore, assessment of clinical utility is essential in the context of automatic FoV prescription, as it determines whether model predictions are not only quantitatively concordant with reference annotations but also sufficiently accurate and diagnostically relevant for practical clinical use. The clinical utility of these predictions was deemed to be high by all five raters, with accuracies above 95%. Across all datasets, the reasons for non-acceptable predictions included three cases with missing predictions, one case with incorrect slice selection, one case with incorrect angulation, and up to five cases with misplaced FoV. In the latter cases, the prostate was enlarged due to underlying pathology. A strategy to improve models’ performance could be to add more diverse augmentations during training. For example, in our study we only used flip and rotate augmentations, but domain-dependent augmentations such as adding noise, artefacts, or changing the overall contrast could further improve the generalizability of our model.

DL-based methods for FoV positioning have been investigated in a limited number of anatomies outside of the prostate. Blansit et al. demonstrated the feasibility of using DL to localize cardiac landmarks for prescribing short-axis, four-chamber, three-chamber, and two-chamber view planes. The model performed well, even though the accuracies varied between the view planes^23^. Allen et al. demonstrated the feasibility of using a network to automatically place scan and pre-scan volumes in breast MRI; the predicted volumes showed a high overall agreement with the human-placed volumes.^24^ Lei et al. proposed a DL framework for automating the FoV prescription step in pediatric abdomen and pelvic MRI scans, using localizer images. While their proposed CNN-based model achieved a 92% acceptance rate for clinical scans, it does not support oblique ROI and FoV prediction^25^. Quinsten et al. demonstrated oblique FoV planning for shoulder MRI using YOLOv11-OBB, with performance noninferior to interrater variability across sites and metrics^29^. For whole-body MRI FoV prescription based on DL segmentations, they reported a mean DSC of 0.98 and high clinical acceptability (98.3% internal, 87.5% external) as assessed by two radiologists and two radiographers^15^.

In contrast to other studies, ours is a multicenter study involving large training and testing datasets. We deliberately employed publicly available data for training to enhance reproducibility. The most crucial difference between our model and those of previous studies is that our model can predict oblique FoVs. Thus, our model could be used in routine clinical work as a supportive tool in the first step as it has been tested on a large cohort including multiple scanners and magnetic field strengths. In addition, we did not exclude scans due to pathologies or artifacts; for instance, images with catheters inserted and radical prostatectomy, which are common in routine clinical work, were present in the data. Changes in the prostate parenchymal environment following biopsy, spacer placement, prostatitis and other factors could affect the computational abilities of the model for accurate FoV planning. Nevertheless, the implementation of our model in clinical practice is constrained by the occurrence of error cases. A comprehensive examination of these cases did not indicate any systematic error. However, as most erroneous cases were associated with pathological enlargement of the prostate and infiltration of adjacent anatomical structures, the inclusion of a larger number of such cases may further improve model performance.

Our study had further limitations. The PICAI dataset we used for training contained only high quality images and was acquired on scanners from two vendors. This may limit the generalizability of the model in clinical routine. Especially in data where the prostate and rectum cannot be clearly delineated, the network might perform worse due to the lack of such data in the training dataset. In addition, our cohorts were collected only from European countries. To enhance the model’s generalizability, we plan to incorporate datasets from non-European regions and a wider variety of scan qualities in future iterations. Additionally, we aim to gather demographic and ethnic data for more comprehensive performance analysis, and will explore data augmentation and domain adaptation strategies to better address variations in scan quality and regional differences. Finally, given the flexibility outlined by PI-RADS v2.1 guidelines and the studies of Caglic and Barrett^12^, Giganti et al.^30^ and Engels et al.^13^ regarding the positioning and angulation of the FoV and no evidence of potential superiority of one approach over another exists we use one of the recommended orientations of the axial and coronal plane in our study as suggested by Engels et al.

Future work should focus on prospective integration of the proposed method into the clinical workflow and on evaluating its performance in real-time routine practice. Although technical integration into vendor-specific scanner software is currently limited by regulatory and technical constraints, the short inference time of less than 10s for both planes compared to 40.6 ± 3.5s for manual FoV prescription and the observed reduction in planning time indicate substantial potential for workflow acceleration. Further studies should also investigate deployment in larger multicenter settings and across different scanner platforms to assess practical feasibility and generalizability.

In conclusion, our multicenter study demonstrates that deep learning-based automated FoV positioning for prostate MRI is both feasible and clinically reliable, achieving accuracy comparable to experienced radiographers across diverse external cohorts. The model’s ability to predict oblique FoVs and its robustness against common clinical imaging variations highlight its potential as a valuable tool to enhance workflow efficiency, reproducibility, and diagnostic consistency. This approach can reduce radiographer workload and support less experienced operators in achieving high-quality imaging. Despite limitations in training data diversity, future enhancements are expected to improve generalizability, positioning this method as a valuable tool for standardized prostate MRI acquisition in clinical practice.

## Methods

### Ethic statement

This retrospective multicenter study was approved by the local ethics committee (Medical Faculty, University Duisburg-Essen; registry number 22-10740-BO). Due to the retrospective nature of the study, Institutional Review Board of the Medical Faculty, University Duisburg-Essen waived the need for obtaining informed consent. This study follows all relevant guidelines and regulations. All data were fully anonymized before being included in the study.

### Patient cohorts

Three cohorts between 2019 and 2024 were collected retrospectively; the publicly available Prostate Imaging: Cancer AI (PI-CAI) dataset^31^ was used for training and validation. Subsequently, three external anonymized cohorts for testing were collected consecutively from three hospitals (Site I, Site II and Site III).

Prostate MRI examinations were included when a corresponding T2WI sagittal scan was available. Scans of patients younger than 18 years were excluded. If a patient had multiple scans, only one was included via random selection. Examinations were excluded if delineation of the prostate was not possible due to poor image quality or the prostate not being fully covered. Scans with a pixel spacing of ≥ 1 mm were also removed.

### Imaging data

The T2WI data comprise turbo spin echo/fast spin echo sequences acquired using cartesian sampling techniques. The images were acquired at a slice thickness from 3 to 4 mm with a phased array coil on 1.5T or 3T scanners from multiple vendors with different protocol parameters across different sites (Table 2).

### Annotations of the field of view

The T2WI sagittal slice in which the prostate and rectum could best be delineated was selected. The positioning and alignment of the oblique coronal and axial planes were performed per the recommendations of Engels et al.^13^ A board-certified radiologist (A.W, with 15 years of experience) and a radiographer (A.S.Q., with 20 years of experience) in consensus performed the ground truth annotations of all cohorts. The annotations were performed using a simple in-house tool developed in Python.

### Intra- and inter-rater variability

The intra- and inter-rater variability in the selected slice, intersection over union (IoU), and mean absolute angle difference (MAAD) were computed because the annotations may vary without affecting the clinical utility. In addition, to judge the relative placement of the FoVs, the center offset and the 95th percentile HD were computed, both relative to the longer side of the ground-truth FoV to account for pathological variations in prostate size. To assess the intra-rater variability, one rater repeated the annotations twice, each after a wash-out period of 1 month, on a set of 100 randomly chosen MRI scans from the test cohort of Site I. Regarding the inter-rater variability, two raters were asked to annotate images independently using a similar approach. Further details are included in the supplemental materials.

### Neural network training

**Fig. 6:**
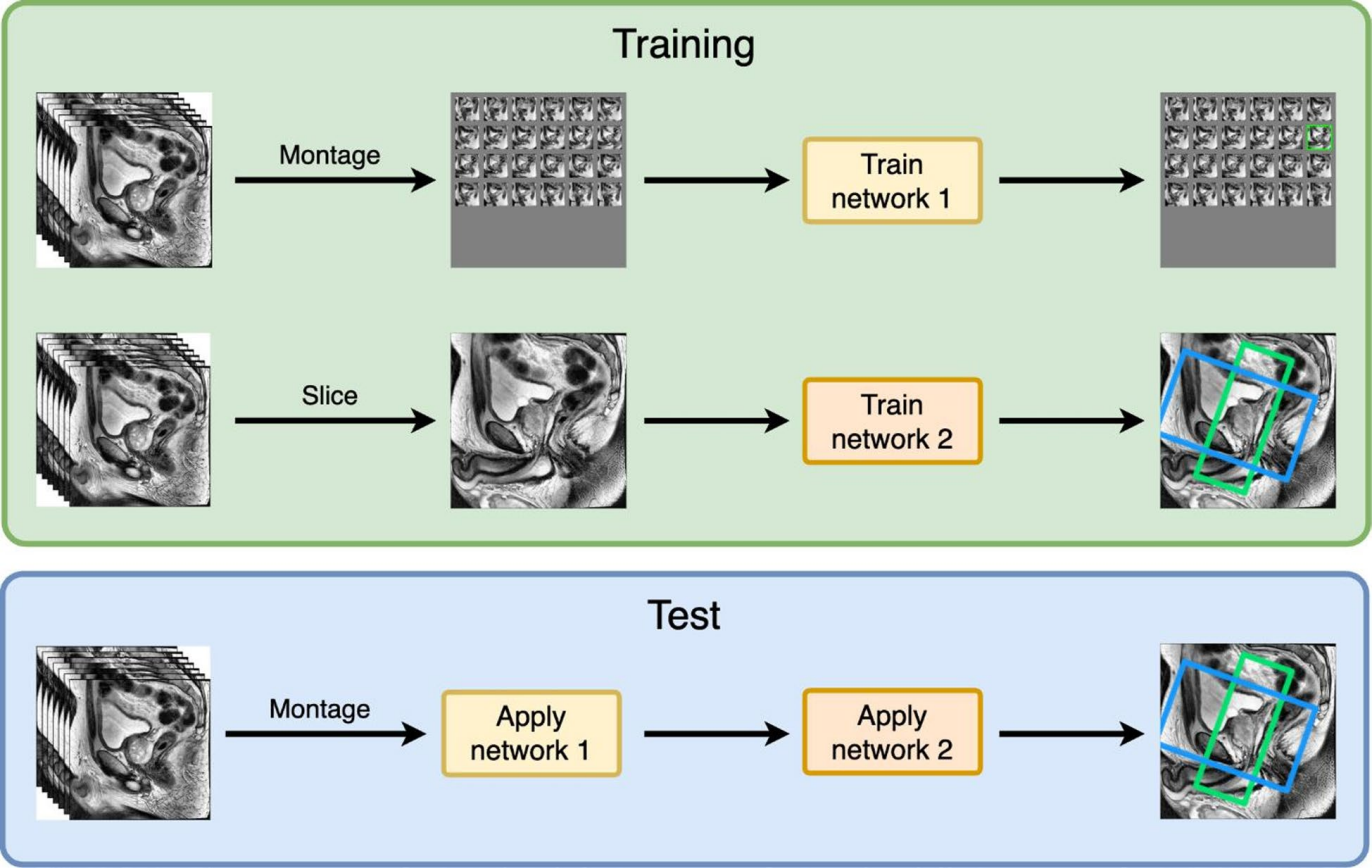
Illustration of the two network stages. The T2WI sagittal slices are first represented as two-dimensional overview/montage images on which the first network is trained to predict the slice on which the prostate is most visible for positioning the FoV (green). The second network is trained to predict the FoV annotation from the best slices. Both networks are trained independently. For predictions on new data, the networks are used consecutively: in the first stage, the first network selects the slice for the FoV annotation; this is then passed to the second network, which predicts the FoV annotation.

The training was performed in two stages (Fig. 6). In the first stage, the network was trained to identify the optimal T2WI sagittal slice for planning, and in the second stage, another network was trained to predict the oblique coronal and axial FoVs on a given T2WI sagittal slice. The training in the second stage was performed independently of the first (i.e., the second network was not trained on the output of the first stage but with the ground truth annotation and slices).

Three commonly used architectures for detecting arbitrarily-oriented objects were applied in both stages: the Rotated RetinaNet^32^, ROI transformer^33^, and ReDet networks^34^. These networks were selected since they demonstrated the highest performance in the MM Rotate model zoo^35^ and ensured accurate detection and alignment across diverse orientations. The networks were used with a ResNet-50 backbone, pre-trained on the Dataset for Object Detection in Aerial Images (DOTA-v1.0)^36^.

Both stages were trained similarly, using a 5-fold cross-validation (CV) during which only the learning rate was optimized. During training, flip and rotation augmentations were applied. The absolute minimal difference in slice position was determined in the first stage. The mean IoU and MAAD were computed in the second stage. The optimal model was that with the lowest mean error across all CV folds.

The training was performed using Python, PyTorch, and the MMRotate framework v0.3.4^37^; further details are included in the supplemental materials. Code will be uploaded to github upon acceptance.

### Testing

The five models trained during CV were used as an ensemble to obtain a prediction on the test set. The median of the five predicted slice positions was used for the first stage. The oblique coronal and oblique transversal FoVs with the highest IoU were selected for the second stage, corresponding to a “geometrical median.”

Predictions on new data were generated by applying the two steps consecutively. For a given T2WI sagittal scan, the first stage was used to select the best slice. Then, the slice was extracted and employed in the second stage. The differences in the selected slice mean IoU, and MAAD were computed and compared with the ground truth. Moreover, absolute differences to the maximum of the observed inter-rater variability were compared via a non-inferiority test. Furthermore, the HD was calculated between the model-predicted FoV and the ground truth^38^.

### Clinical utility

A well-established method for evaluating the diagnostic usefulness of model-predicted FoV prescriptions is clinical utility assessment^15,39,40^. Therefore, the network’s predictions were qualitatively judged by five readers. A prediction was considered clinically useful if it allowed for diagnosis. This was the case when the entire prostate, irrespective of size and shape, and the seminal vesicles were included in the FoV, as per PI-RADS v2.1. Additionally, FoV encompassing a minimum of two-thirds of the seminal vesicles was also considered clinically useful, aligning with the recommendations by Engels et al. and consistent with prevailing clinical practices.^13^ Cases that were deemed to fail the clinical utility assessment were classified into four categories: wrong slice selection, incorrect angulation, misplaced prediction, and missing prediction.

### Sensitivity analysis

The robustness of the model’s second stage was tested on 30 randomly selected T2WI sagittal slices from each center. The images were modified by adding Gaussian noise of varying strengths and, separately, via increasing contrast levels by linearly stretching the intensity range.

### Network-rater variability

To assess the model within the context of expert variability, we evaluated the network as an additional independent rater. To ensure comparability, we restricted the analysis to cases where the network selected the same slice as the ground truth. We calculated the MAAD, IoU, center offset, and HD95 between network predictions and each human rater, providing a direct comparison of model performance against the observed range of expert variability.

### Statistical analysis

The age differences between the two cohorts were assessed using t-tests. Descriptive values were reported as the mean ± standard deviation (SD). A sample size analysis for a one-sided t-test with a power of 0.9 and a significance level of 0.025 was conducted for non-inferiority testing. Assuming that a difference of 2.5**°** in the angle, 0.15 in the IoU, and 2.5% in the two distance measures would not lead to inferior FoVs, the calculations yielded a minimum sample size of 44. Significant differences were set at *P* < 0.05. No adjustments were performed for multiple tests. The statistical analyses were conducted using Python 3.8.

## Supplementary Information

Below is the link to the electronic supplementary material.

Supplementary Material 1

## Data Availability

The source code is publicly available at: https://github.com/aydindemircioglu/prostate.FOV. The trained model can be obtained from Zenodo at: https://zenodo.org/records/20082586. The PI-CAI training dataset is also available via Zenodo at: https://zenodo.org/records/6624726.
