# Supplementary material for: Deep learning-based automatic field of view planning for prostate MRI in oblique coronal and oblique axial planes
Source: Sci Rep. 2026 May 11;16:14731. doi: 10.1038/s41598-026-52248-6 (PMC13161440; doi:10.1038/s41598-026-52248-6)
Supplement: Supplementary file 1 — Supplementary Material 1 [file 41598_2026_52248_MOESM1_ESM.docx]

### **Appendix E1**

#### Patient cohorts

The publicly available Prostate Imaging: Cancer AI (PI-CAI) dataset contains 1500 anonymized prostate MRI scans from 1476 patients, acquired between 2012 and 2021, at three centers (Radboud University Medical Center, University Medical Center Groningen, and Ziekenhuisgroep Twente) based in the Netherlands. All patients were included; however, only one scan (chosen randomly to avoid bias) was retained per patient. The T2WI sagittal scans used in this study were unavailable for two patients, resulting in a cohort of 1474 scans.

An external anonymized cohort for testing was collected by querying the radiological information system (RIS) and the picture archiving and communication system (PACS) at Site I. All prostate MRI examinations between January 2011 and December 2022 were included when a corresponding T2WI sagittal scan was available.

A second external anonymized test cohort was collected from Site II by randomly selecting 50 prostate MRI scans with a T2WI sagittal scan. The same inclusion and exclusion criteria were applied, resulting in a cohort of 46 scans.

Finally, a third anonymized test cohort was collected from Site III. A total of 60 prostate MRI scans were randomly selected, 58 of which were included in the study.

Altogether, 1474 prostate MRI scans for training and 530 for external testing were included.

#### Prostate MRI scanning procedure

The prostate MRI scanning procedure begins with a pilot scan, which provides an overview of the pelvis. In addition, a T2WI sagittal image of the prostate is usually acquired based on the pilot scan. Radiographers then use the T2WI sagittal image to visually determine the location and orientation of the FoV in different planes for high-resolution T2WI, DWI, and DCE according to PI-RADS guidelines. The first step involves selecting the slice in the sagittal T2WI in which the prostate is best delineated. The FoV is then precisely positioned and angled on the selected optimal slice. The stack of slices is traversed to check whether the prostate and seminal vesicles are completely covered. The other orientations are then used to ensure that the prostate and seminal vesicles have been fully captured and adjusted such that no aliasing artifacts can occur. Either an endorectal or phased array coil can be used. The dataset presented in this study comprises images acquired solely with phased array coils at different magnetic field strengths (1.5T and 3T) on scanners from multiple vendors across different centers.

#### Intra- and inter-rater variability

Given that the annotations may vary without affecting their clinical utility, the intra-rater and inter-rater variability in the selected slice, the mean overlap and angle difference were computed. To assess the intra-rater variability, one rater (A.S.Q.) repeated the annotations twice, each after a wash-out period of one month, on a set of 100 randomly chosen MRIs from the test set of site I. However, as the annotations may occur on different slices, and these are not directly comparable due to anatomical differences, the rater was first asked to select the best slice for annotation in each scan. Subsequently, following another wash-out period of one month, the rater was presented with the slices of the ground-truth annotation and was then asked to annotate them. From these annotations, two measurements were computed: a) the mean difference to the best slice (measured in slices), and b) the mean absolute angle difference in the FoV (MAAD) and the mean overlap in terms of Intersection over Union (IoU).

To assess inter-rater variability, two raters (A.S.Q. and M.D.), and a radiographer (with five years of experience in prostate MRI) were asked to independently annotate the 100 T2WI images in a similar manner. This entailed first selecting the optimal slice for annotation and then, following a wash-out period, annotating the FoVs in the ground-truth slice. Once more, the difference in selected slice, the mean overlap, and angle difference were calculated.

#### Preprocessing

The scans were converted from 16-bit grayscale to 8-bit pseudo-RGB by dividing by 256 and applying three different contrast-limited adaptive histogram equalization (CLAHE) filters^1^ with varying strength to the image. Specifically, the image was equalized with a clip limit of 64 and a block size of (1, 1), a clip limit of 32 and a block size (2, 2), and a clip limit of 16 and a block size of (4, 4). The resulting images were then used as red, green, and blue channels to form the RGB image used for training and prediction. Subsequently, the images were rescaled to a size of 1184x1184 and normalized to the intensity mean and standard deviation of the dataset for Object Detection in Aerial Images (DOTA-v1.0).^2^

#### Training stages

Training was conducted in two stages using 2D networks. The rationale for adopting this approach over a 3D network trained in an end-to-end manner is as follows: First, given the relatively limited sample sizes, a 3D network may exhibit suboptimal performance. Second, there is currently no comprehensive database comprising such 3D images and annotations that could be leveraged for pretraining. Third, there is currently no widely used 3D network architecture that is capable of detecting rotated objects in 3D. Therefore, developing a custom 3D network architecture would entail extensive development with unclear benefits. Using commonly used 2D network architectures that are easily available and have been shown to perform well was regarded as a more reasonable alternative.

The two stages were as follows: In the initial stage of the process, the network was trained to identify the optimal T2WI sagittal slice. In the subsequent stage, a second network was trained to predict the oblique coronal and axial FoV on a given T2WI sagittal slice.

In the initial stage, all sagittal slices of the T2WI scan were rescaled to a uniform width of 160. This width was chosen heuristically. The slices were then montaged into a single image with a padding of 128 pixels (Fig. 2). The padding was included to facilitate the selection of a specific slice by the network. Without padding, the network would first need to learn to understand where merged slices begin and end. The slices were merged using a 6 by 6 grid, resulting in a total of 36 slices per montaged image. This number was chosen based on experience and informed by the acquisition parameters, which included slice thicknesses between 3 and 4 mm and the overall scan size. If fewer slices were available, the slices were pasted at a random start position, ensuring the presence of empty slices at the beginning and end of the montaged images. In the case that a greater number of slices were available, only those situated in the middle were selected for the montage, with the slices at the top and bottom being discarded. This is a reasonable approach, given that in clinical practice the prostate is typically situated in the middle of the sagittal T2WI. The montaged images were then annotated by a non-oblique rectangle around the ground-truth slice. Training was then performed to predict these annotations.

In the second stage, a second network was trained to predict the oblique coronal and axial FoV on a given slice. In this step, for each T2WI sagittal scan, three slices were annotated: the best slice and, where possible, the two bordering slices. This approach was taken primarily because these two slices can, in most cases, be equally well used for the annotation. This is further underlined by the observed intra-rater variability. The annotation of these two additional slices increased the overall sample size by three, which is beneficial for network training. It should be noted that this technique to increase the sample size was not applied in stage 1.

The training of the second stage was performed independently of the first stage. This means that the second network was not trained on the output of the first stage, but rather with the ground truth annotation and slices. Therefore, any errors in the first stage did not directly propagate to the second stage network.

For both networks, three commonly used architectures were employed: The Rotated RetinaNet^3^, the ROI transformer^4^, and the ReDet networks^5^. Implementations from the MMRotate framework (v0.3.4) were utilized^6^. These networks were selected since they demonstrated the highest performance in the MMRotate model zoo. The networks were utilized with a ResNet-50 backbone and were all pretrained on the DOTA-v1.0 dataset.

#### Network training

Both stages were trained using a similar methodology. A 5-fold cross-validation (CV) approach was employed during training. The batch size was selected to be as large as possible, resulting in a size of 8. All parameters were left at their default values, with the exception of the learning rate, which was chosen between 0.08, 0.04, 0.02, 0.008, and 0.004. The rationale for this approach is that the learning rate is regarded as the most crucial hyperparameter. Optimizing other hyperparameters could potentially enhance overall performance, but would exponentially increase training times. During training, the mean average precision of the Intersection-over-Union (IoU) (across the range of 0.50 and 0.95) was utilized as a loss metric to assess model performance. The training proceeded for 12 epochs, which is the default value that was used during the pretraining of the networks on the DoTA dataset in the MMRotate framework. The stochastic gradient descent (SGD) optimizer was employed, with a momentum of 0.9 and a weight decay of 0.0001. The learning rate was scheduled with a linear warmup of 500 iterations, and multiplied at epochs 8 and 11 by 0.1. During training, a limited number of augmentations were applied to the images to synthetically increase the sample size, as it is known that networks perform better with larger sample sizes. Therefore, the images were rotated horizontally and/or vertically with a probability of 25%. In addition, the images were also rotated with a probability of 50% with a random degree between -20 and 20 degrees for the first stage and with a random degree for the second stage. After training, the performance of the networks was evaluated on the validation fold. Training was conducted on a workstation equipped with an Intel Core i9-9900K, 128 GB of RAM, and an NVIDIA GeForce RTX 3080 graphics card with 10 GB of VRAM. All experiments were conducted on a Ubuntu 22.04.3 LTS operating system.

#### Network training results

Training was completed successfully in both stages. Due to the reduced learning rate, the network converged after twelve epochs and showed high performance on the validation set.
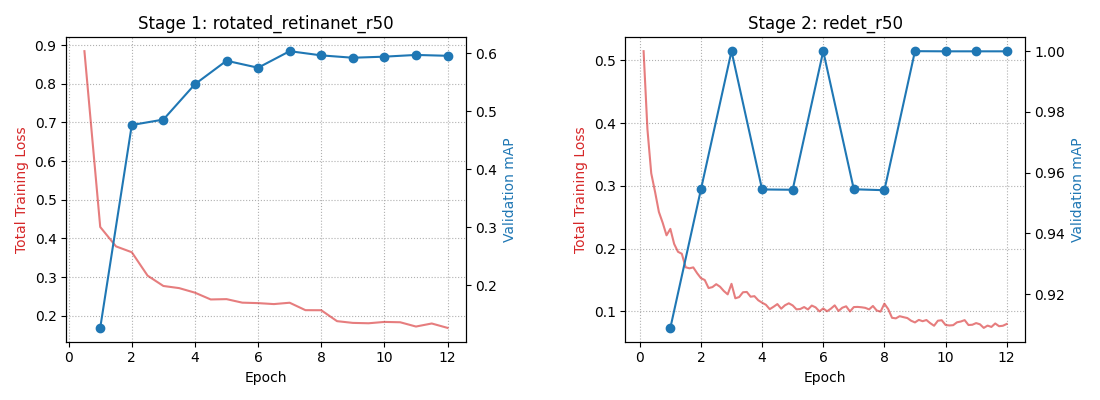


#### Network validation

In the initial stage, the minimal difference in slice position was computed. For instance, if the selected slice was nine, and the network selected seven, then an error of two was made. The model with the lowest mean overall error across all CV folds was selected as the best-performing model.

In the second stage, two metrics were computed: the mean Intersection-over-Union (IoU) and the mean absolute angle difference (MAAD). The IoU was computed as the mean of the IoU of the predicted oblique coronal and axial field-of-views (FoV) with the corresponding ground truth FoVs, while the MAAD was computed as the mean angle between them. Subsequently, both scores were combined heuristically into a single error measure by computing E = IoU[in %] + 2*(90 - AAD). This metric counts errors in MAAD approximately 1.8 times more frequently than in IoU. This was chosen since an error in MAAD is regarded as more severe than an error in overlap, given that a different angle can result in scans where the prostate appears differently than expected (Figure E1). However, a difference in overlap will not affect the visual quality, but the visible area.

Given that both oblique focal planes must be orthogonal to one another, and that the networks did not necessarily ensure this, a simple post-hoc fix was applied to the output of the second-stage networks. The oblique coronal focal plane was fixed, and the oblique axial focal plane was rotated such that it was fully orthogonal. This resulted in focal planes following the PI-RADS guideline.

All utilized network models were capable of making multiple detections. In this instance, the network with the highest confidence was selected. The optimal network architecture with regard to this metric was then chosen as the final model.

*Network-Rater Variability*

After training was finalized, the network was evaluated as an independent rater to benchmark performance against human variability. To ensure valid comparison, only cases where the network correctly selected the ground-truth slice were included. This resulted in 65 cases being analyzed. In these instances, network predictions were treated as an additional rater and compared against the three human observers. Angular difference, Intersection over Union (IoU), center offset, and 95th percentile Hausdorff Distance (HD95) were calculated for these comparisons. The results were as follows:

**MAAD Angle (degree)**

Lower triangle = coronal, upper triangle = transversal

|  | Rater 1 | Rater 2 | Rater 3 | NN |
| --- | --- | --- | --- | --- |
| Rater 1 |  | 2.85 ± 4.33 | 5.73 ± 5.06 | 3.22 ± 3.31 |
| Rater 2 | 2.79 ± 4.31 |  | 5.93 ± 5.50 | 3.09 ± 2.85 |
| Rater 3 | 5.73 ± 5.05 | 5.88 ± 5.51 |  | 5.23 ± 4.33 |
| Network | 3.25 ± 3.35 | 3.07 ± 2.80 | 5.29 ± 4.37 |  |

**IoU**

Lower triangle = coronal, upper triangle = transversal

|  | Rater 1 | Rater 2 | Rater 3 | NN |
| --- | --- | --- | --- | --- |
| Rater 1 |  | 0.86 ± 0.09 | 0.71 ± 0.08 | 0.76 ± 0.07 |
| Rater 2 | 0.87 ± 0.08 |  | 0.69 ± 0.07 | 0.75 ± 0.08 |
| Rater 3 | 0.72 ± 0.07 | 0.73 ± 0.08 |  | 0.67 ± 0.07 |
| Network | 0.80 ± 0.05 | 0.80 ± 0.06 | 0.73 ± 0.07 |  |

**Center offset (%)**

Lower triangle = coronal, upper triangle = transversal

|  | Rater 1 | Rater 2 | Rater 3 | NN |
| --- | --- | --- | --- | --- |
| Rater 1 |  | 3.32 ± 2.70 | 10.57 ± 4.16 | 3.71 ± 2.38 |
| Rater 2 | 2.74 ± 1.87 |  | 11.32 ± 3.58 | 3.39 ± 2.68 |
| Rater 3 | 6.32 ± 2.07 | 5.94 ± 2.13 |  | 11.55 ± 2.58 |
| Network | 3.46 ± 2.30 | 3.53 ± 2.41 | 4.87 ± 2.20 |  |

**95 percentile Hausdorff distance (%)**

Lower triangle = coronal, upper triangle = transversal

|  | Rater 1 | Rater 2 | Rater 3 | NN |
| --- | --- | --- | --- | --- |
| Rater 1 |  | 6.74 ± 5.27 | 15.14 ± 5.16 | 10.59 ± 4.67 |
| Rater 2 | 5.49 ± 5.07 |  | 16.02 ± 4.94 | 10.60 ± 4.88 |
| Rater 3 | 12.02 ± 4.51 | 11.77 ± 5.25 |  | 18.28 ± 3.61 |
| Network | 9.08 ± 3.89 | 9.11 ± 3.93 | 11.93 ± 4.32 |  |


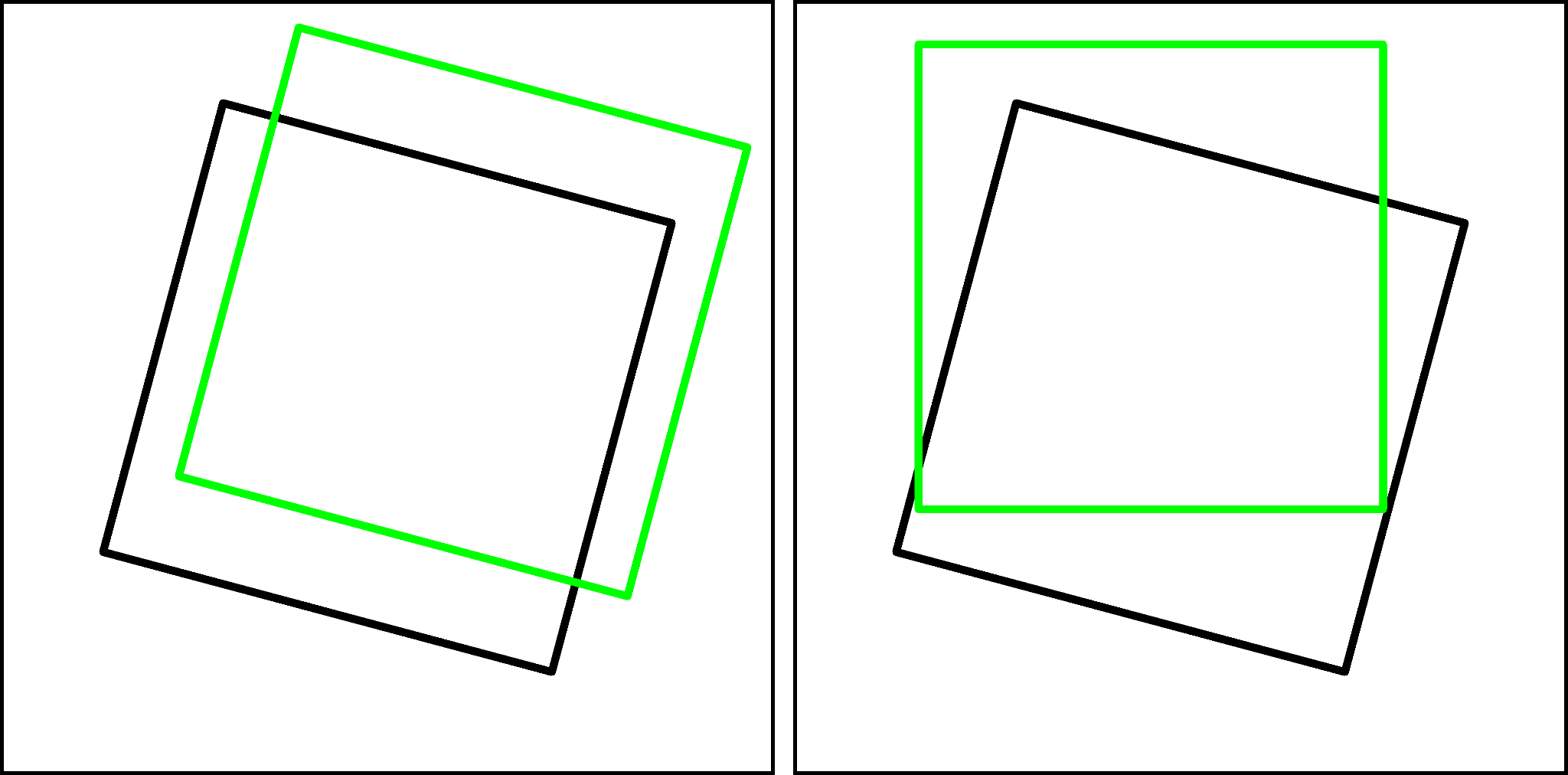


**Figure E1**: Plot of a set of boxes with the same overlap. The ground truth is marked in black, the prediction in green. On the left plot, the prediction is only shifted, and in the resulting axial and coronal scans, the prostate will appear similar to that in the ground truth, albeit with a different crop. On the right plot, however, the prediction is rotated with respect to the ground truth box. This will result in scans where the prostate might not be in a heart shape, or where the axial scan might not be perpendicular and orthogonal to the rectum. Therefore, although both predictions have the same overlap, the left prediction can be regarded as superior to the right one.

**References**

1. Pizer, S. M. *et al.* Adaptive histogram equalization and its variations. *Computer Vision, Graphics, and Image Processing* **39**, 355–368 (1987).

2. Ding, J. *et al.* Object Detection in Aerial Images: A Large-Scale Benchmark and Challenges. *IEEE Transactions on Pattern Analysis and Machine Intelligence* **44**, 7778–7796 (2022).

3. Zhou, Y. *et al.* MMRotate: A Rotated Object Detection Benchmark using PyTorch. in *Proceedings of the 30th ACM International Conference on Multimedia* 7331–7334 (Association for Computing Machinery, New York, NY, USA, 2022). doi:10.1145/3503161.3548541.

4. Ding, J., Xue, N., Long, Y., Xia, G.-S. & Lu, Q. Learning RoI Transformer for Oriented Object Detection in Aerial Images. in *2019 IEEE/CVF Conference on Computer Vision and Pattern Recognition (CVPR)* 2844–2853 (2019). doi:10.1109/CVPR.2019.00296.

5. Han, J., Ding, J., Xue, N. & Xia, G.-S. ReDet: A Rotation-equivariant Detector for Aerial Object Detection. in *2021 IEEE/CVF Conference on Computer Vision and Pattern Recognition (CVPR)* 2785–2794 (2021). doi:10.1109/CVPR46437.2021.00281.

6. Chen, K. *et al.* MMDetection: Open MMLab Detection Toolbox and Benchmark. Preprint at https://doi.org/10.48550/arXiv.1906.07155 (2019).
